# A new species of supergiant *Bathynomus* A. Milne-Edwards, 1879 (Isopoda: Cirolanidae) from the Paracel Islands, South China Sea

**DOI:** 10.3897/BDJ.13.e144238

**Published:** 2025-03-31

**Authors:** Ming-Chih Huang, Tadashi Kawai

**Affiliations:** 1 National University of Tainan, Tainan, Taiwan National University of Tainan Tainan Taiwan; 2 Central Fisheries Research Institution, Hokkaido, Japan Central Fisheries Research Institution Hokkaido Japan

**Keywords:** Cirolanidae, South China Sea, Paracel Islands, *Bathynomusparacelensis* sp. nov.

## Abstract

**Background:**

*Bathynomusparacelensis* sp. nov., a medium-sized supergiant *Bathynomus*, is described from specimens obtained at Zhengbin fishing port in Keelung, Taiwan and had been caught in the water near Paracel Islands, South China Sea. Due to its similar shape to *B.jamesi*, this species has often been mistaken for juveniles or immatures of *B.jamesi* by fishermen working in this area. Species of *Bathynomus* can be distinguished morphologically and genetically. The differences from *B.jamesi* are in the shorter body, clypeus shape, uropod endopod and gene sequence. The difference from *B.vaderi* is in the body shape, clypeus shape, hook number of maxilliped endite and spines number of maxilulla. Based on the morphological and genetic data results, the specimen is a hitherto undescribed species. The samples were collected as a bycatch species in the deep-sea bottom trawl fishery. The distribution area and depth of this new species and population size are still unclear.

**New information:**

*B.paracelensis* sp. nov. is the third supergiant *Bathynomus* discovered in the South China Sea after *B.jamesi* and *B.vaderi*. Its remarkable feature is its short body length and sub-parallel shape. In addition, it is different from *B.jamesi* and *B.vaderi* in features such as clypeus shape, number of maxillula keratinised spine and pleotelson spine almost straight. Phylogenetic and barcoding gap analyses confirm that *B.paracelensis* sp. nov. is not the same species as *B.jamsei*. Many morphological differences also indicate that it should be a different species from *B.vaderi*. *B.paracelensis* sp. nov. may be an intermediate species between giant and supergiant, possessing characteristics of both categories, which can increase researchers' understanding of *Bathynomus* biodiversity.

## Introduction

In recent years, deep-sea environmental protection and biological species research have attracted public attention (Yang 2023). *Bathynomus* is a well-known representative genus of deep water marine life. Many aquariums and museums have launched *Bathynomus* exhibitions to let the public understand *Bathynomus* and the importance of the deep sea in material circulation (Wilson and Ahyong 2015). In Taiwan, the *Bathynomus* ramen launched at a ramen shop in Taipei City in late May 2023 placed the supergiant *B.jamesi* found in the deep South China Sea, directly on the noodle bowl, which attracted public attention. The use of *Bathynomus* as a delicacy is controversial. Food safety experts in Taiwan have also discussed whether food, including toxins or heavy metals, is safe. Ecologists discuss that deep-sea ecology may be disturbed if *Bathynomus* populations decline due to human capture (Everington 2023).

*Bathynomus* was first recorded by A. Milne Edwards in 1879 when he discovered a giant deep-sea isopod in fishermen's nets along the coast of Dry Tortugas in the Gulf of Mexico. Named *Bathynomusgiganteus* (Milne-Edwards 1879, Holthuis and Mikulka 1972), the new species proved the theory of the existence of deep sea creatures. Milne-Edwards (1879) cleverly integrated the meaning of "deep sea family" into the genus name ("*bathy*" means "deep sea" in Latin).

Twenty extant and four fossil species have been described (Huang et al. 2022). Lowry and Dempsey (2006) divided *Bathynomus* into groups, giants and supergiants, according to body size. A giant has a body length of less than 15 cm and a supergiant is described as having a body length greater than 17 cm. Undoubtedly, the epitome of a giant is *B.doederleini* Ortmann. 1894 and the supergiant is typified by *B.giganteus* A. Milne Edwards, 1879.

There are five named species of *Bathynomus* from Taiwan Strait to the South China Sea, including three species of giant *Bathynomus*, *B.affinis* Richardson, 1910, *B.decemspinosus* Shih, 1972 and *B.doederleini* Ortmann, 1894 and two species of supergiant, *B.jamesi* Kou, Chen and Li 2017 and *B.vaderi* Ng, Sidabalok and Nguyen, 2025 (Huang et al. 2022, Ng et al. 2025). Huang and Bruce (2024) discussed the published records of *B.kensleyi* from the South China Sea and concluded that *B.kensleyi* is found only in eastern Australian waters and does not occur in the South China Sea. Lowry and Dempsey (2006) classified a supergiant *Bathynomus* from the South China Sea as *B.kensleyi*, a misidentification (Huang and Bruce 2024). In addition, records of *B.kensleyi*, *B.decemspinosus* and *B.doederleini* from the Indian Ocean (Sankar et al. 2011, PrasannaKumar et al. 2020) are all misidentifications (Huang and Bruce 2024).

The record of the supergiant *Bathynomus* in the South China Sea originated from Soong (1992) and misidentification as a species of *B.giganteus*. Lowry and Dempsey (2006) named the supergiant *Bathynomus* from the South China Sea and the Sea of Eastern Australia a new species of *B.kensleyi*, based on morphological characteristics. Kou et al. (2017) published a new supergiant *Bathynomus* species in the South China Sea, *B.jamesi*. Still, all the samples were immature, not possessing the characteristics a mature individual should have. Huang et al. (2022) suspected a correlation between *B.kensleyi* and *B.jamesi* in the South China Sea. They thought they may be the same species in different development stages. Huang and Bruce (2024) used molecular biology to prove that *B.kensleyi* in the South China Sea was a misidentification of *B.jamesi*.

On 28 January 2023, four female *Bathynomus* specimens, in shape between *B.jamesi* and *B.doederleini*, were collected from the Zhengbin fishing port in Keelung, Taiwan. At first glance, they looked like *B.jamesi* juveniles, but the female individuals were mature, with well-developed oostegites. Morphological and molecular methods were used to confirm the identities of these four specimens as separate, previously unnamed species of *Bathynomus*.

## Materials and methods

### Specimen collection

Specimens (NMMB-CD006299-006302, Table 1) were purchased at the Zhengbin fishing port in Keelung, Taiwan, on 28 January 2023. According to the voyage records, the *Bathynomus* were caught in the South China Sea about 150 km northeast of the Paracel Islands (Xisha Islands in Mandarin, 19.08333 N, 115.25 E). The specimens were taken from waters outside the protected area, preserved in ice aboard the ship and stored at -20°C in the laboratory.

Four *Bathynomus* specimens (Table 1) have been deposited and registered in the National Museum of Marine Biology, Checheng, Taiwan (registration code: National Museum of Marine Biology, NMMB, from NMMB-CD006299-006302).

**Abbreviations**. RS—robust seta/e; TMCD—National Taiwan Museum code, NMMB— National Museum of Marine Biology, Checheng, Taiwan; TL—total length; CL—cephalic length.

### Morphological observations

**Figure 1.**
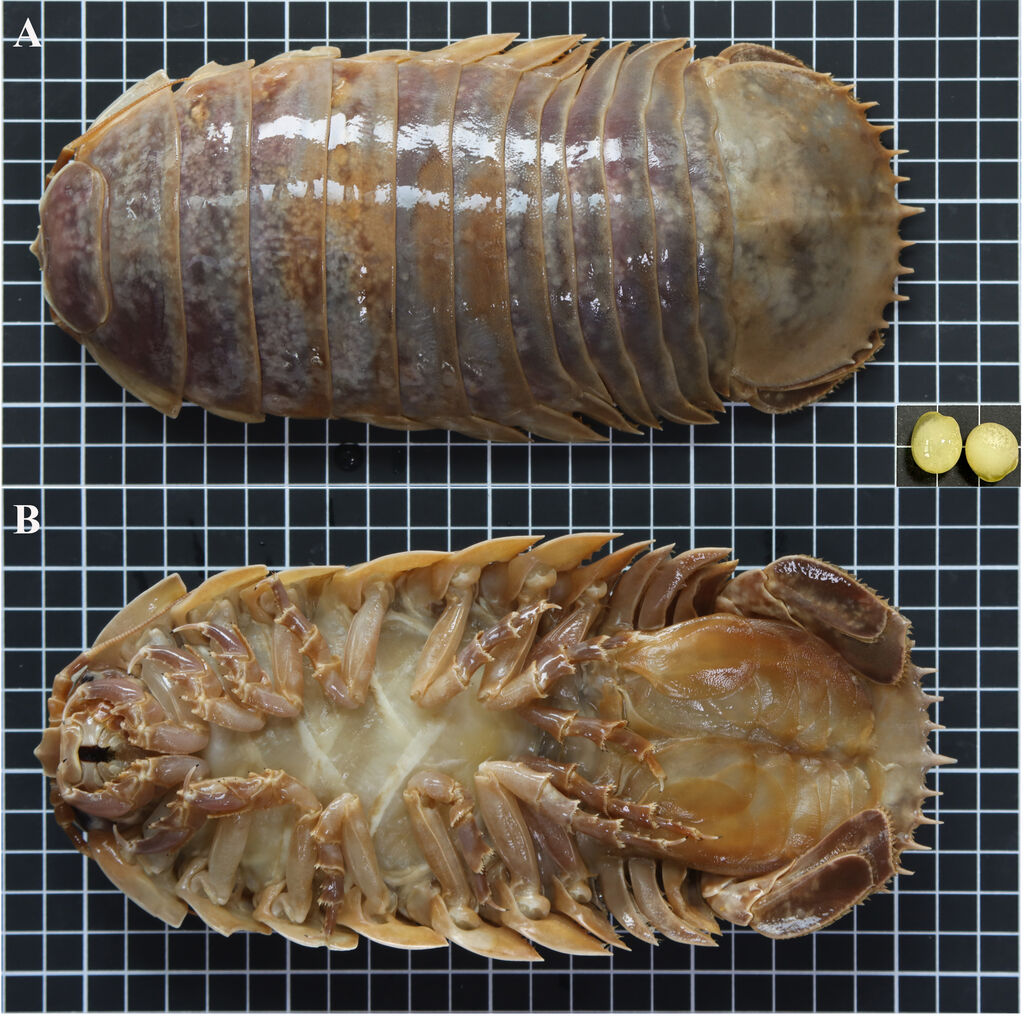
Holotype of *Bathynomusparacelensis* sp. nov. (female, voucher singular: NMMB-CD006300, 220 mm. The South China Sea, the water of Paracel Island (19.0833 N, 115.25 E), coll. Ming-Chih Huang, 28 January 2023). **A** dorsal view, the lower right corner is an ovum, slightly oval and about 12-15 mm in diameter; **B** ventral view. Scale bars: 1 cm.

The specimen and dissected body parts (holotype, voucher singular: NMMB-CD006302, Fig. 1) were photographed using a high-resolution monocular camera (Canon EOS 90D, Tokyo, Japan). A digital camera (Sony MEX-5R, Tokyo) on a stereomicroscope (Olympus, SZ61, Tokyo) was used for illustration. The software CLIP STUDIO PAINT (https://www.clipstudio.net/ja/functions/) and Adobe Illustrator CS (Adobe Inc., San Jose, CA, USA) were used to make a line drawing illustration from an image file of a digital camera. The observed specimens were measured using a stereomicroscope's eyepiece micrometer. Dimensions from the measurements were labelled on each illustration. The terminology of body parts follows Huang et al. (2022). Total length (TL) and cephalic length (CL) were measured from the anterior tip of the head to the posterior end of the pleotelson plate with a central spine, respectively.

### Molecular biological analysis

Total genomic DNA of *Bathynomus* samples (NMMB-CD006299-006302) were extracted from ca. 25 mg of pereopod muscle resected from four specimens from off Paracel Islands waters, using a commercial genomic DNA extraction kit (QIAamp DNA Mini Kit, Hilden, Germany) according to the manufacturer’s protocol. PCR primers used for the amplification were designed, based on the sequences of the genes encoding *COI* (Folmer et al. 1994) and 16S ribosomal RNA (Palumbi et al. 1991). In addition, we also used our own designed primers as Kmae (F) and Kushi (R) for the test (Table 2). The four samples were all sequenced for *COI* and 16S rRNA (Table 1).

Amplification using the *COI* and 16S rRNA primers was based on a cycle of denaturation at 94ºC for 30 s, annealing at 48ºC for 40 s and extension at 72ºC for 30 s using a DNA thermal cycler model MyCyclerTM Thermal Cycler System (#1709703, Bio-Rad, Hercules, CA, USA). This procedure was carried out for 35 cycles and the final extension step was performed at 72ºC for 10 min. The 100 μL reaction medium contained 200 nM dNTPs, 10 mM each of forward and reverse primers, 2 units of Ex-Tag DNA polymerase (TaKaRa Ex Taq® DNA Polymerase, Takara Bio, Shiga, Japan), 10 μL of 2× Ex-Tag DNA polymerase buffer (Takara Bio) and 50 ng of genomic DNA. The PCR products were subjected to electrophoresis using 1% agar (VWR Funding Inc, West Chester, PA, USA) and visualized with SYBR Green (HealthView Nucleic Acid Stain, Thermo Fisher Scientific, Waltham, MA, USA). After confirming the success of PCR amplification, the products were sent to Biotech (Genomics, Xizhi District, New Taipei City, Taiwan) for sequencing. The obtained sequences were edited and aligned using editing software BioEdit 7.2 (https://bioedit.software.informer.com/7.2/) and Multiple Sequence Alignment (Clustal Omega – GenomeNet, Hinxton, Cambridgeshire, UK).

### Phylogenetic and Barcoding gap analyses

Comparisons of the edited and aligned *COI* and/or 16S rRNA sequences of the present specimens and seven reported sequenced species of *Bathynomus* were performed using Molecular Evolutionary Genetics Analysis 11 (MEGA 11) software (Tamura et al. 2021). *COI* sequence data were obtained from the National Center for Biotechnical Information (NCBI) for *B.jamesi* (KX417647) (from the sea off the southern part of Hainan Island, China, Kou et al. (2017)), *B.kensleyi* (OQ860751) (from Marion Plateau, Coral Sea, QLD, Australia, Huang et al. (2022)), *B.yucatanensis* (MZ354630) (from the Gulf of Mexico off the Yucatan Peninsula, Huang et al. (2022)), *B.giganteus* (MG229639) (from the northern Gulf of Mexico, except for De Soto Canyon, Timm et al. (2018)), *B.maxeyorum* (KT963292) (from Bahamas, West Atlantic, Shipley et al. (2016)), *B.kapala* (OQ970652) (from eastern Australian waters, unpublished) and *B.doederleini* (MZ723938) (from Sagami Bay, Japan, unpublished). *B.decemspinosus* from the Indian Ocean is registered in NCBI, but is considered a misidentification (Huang and Bruce 2024). 16S rRNA sequences for *B.jamesi* (KX417643) (from the sea off the southern part of Hainan Island, China, Kou et al. (2017)), *B.kensleyi* (OQ865221) (from Marion Plateau, Coral Sea, QLD, Australia, Huang et al. (2022)), *B.yucatanensis* (MZ042927) (from the Gulf of Mexico off the Yucatan Peninsula, Huang et al. (2022)), *B.giganteus* (MG229479) (from the northern Gulf of Mexico, except for De Soto Canyon, Timm et al. (2018)) and *B.doederleini* (OR239864) (from Suruga Bay, Japan, unpublished) were obtained. The nucleotide sequence for Cirolanidae (*Atarbolanaexoconta* Bruce and Javed, 1987) *COI* (KX782999) and (*Excirolanahirsuticauda* Menzies, 1962), 16S rRNA (MK898194) were used as the outgroup control.

Using Drawtree (Phylip software package, http://bioweb.pasteur.fr/seqanal/interfaces/drawtree.html), phylogenetic trees were constructed by the Neighbour-joining (NJ) method under some different techniques (Nei and Kummer 2000). The percentage of replicate trees where the associated taxa clustered together in the bootstrap test (1000 replicates) is shown above the branches. Using Kimura 2 parameters (K2P) genetic distance (Kimura 1980) in MEGA 11, pairwise distance analysis was carried out (Tamura et al. 2007, Tamura et al. 2021).

## Data resources

### Taxonomy

**Order Isopoda Latreille, 1989**

**Family Cirolanidae Dana, 1852**

**Genus *Bathnomus* A. Milne-Edwards, 1879**

**Comparative material**

*Bathynomusjamesi* Kou, Chen and Li, 2017, male (TMCD003327), TL 355 mm, CL 197 mm, waters of the South China Sea about 300 km southwest of Pratas Island, 19.084N, 115.250E, bottom trawl, depth was about 420-550 m. 12 May 2020. *Bathynomusgiganteus* A. Milne-Edwards, 1879, male (TMCD003336) exchanged with Japan's Shin Enoshima Aquarium (Fujisawa, Kanagawa, Japan), TL 316 mm, CL 172 mm, baited cage at a depth of 600-800 m on 19 April 2017 in the Gulf of Mexico off the Yucatan Peninsula. *Bathynomusyucatanensis* Huang, Kawai and Bruce 2022, holotype (TMCD003335) exchanged with Japan's Shin Enoshima Aquarium (Fujisawa, Kanagawa, Japan), male, TL 257 mm, CL 129 mm and wet weight 550 g, baited cage at a depth of 600-800 m on 19 April 2017 in the Gulf of Mexico off the Yucatan Peninsula. *Bathynomusdoederleini* Ortmann, 1894, specimens (NMMB-CD003011), TL 128 mm, CL 47 mm, 28 Aug 2008, 122°2.751E. 24°53.324N, off Tai-chi, I-lan County, Taiwan, depth 600 m.

## Taxon treatments

### Bathynomus paracelensis sp. nov.

8CC82366-3695-508D-B3A1-9A6F9996DAA8

9A179F2D-AF03-4542-A969-467535639D5F

PP715921

PP715922

PP715923

PP715924

PP719187

PP719188

PP719189

PP719190

**Restricted synonymy**: A. Milne-Edwards, 1879: 21; Bruce, 1986 126: Kensley and Schotte 1989: 129; Soong, 1992:293, figs. 1, 2, Lowry and Dempsey, 2006: 184, figs. 18, 19; Kou, Chen and Li 2017:285, figs. 2-7; Huang, Kawai and Bruce, 2022: 890, figs. 3-7.
**Type species**: *B.giganteus* A. Milne Edwards, 1879; by monotype (Bruce 1986).

#### Materials

- **Type status:** Holotype. **Occurrence:** occurrenceRemarks: bottom trawl by the crew of Keelung-based fishing vessel Jing yang; recordedBy: Ming-Chih Huang; sex: female; lifeStage: adult; preparations: whole animal; associatedSequences: Gene Bank: PP715922, PP719190; occurrenceID: A48C2F53-9A55-5317-81A0-A67C34838E86; **Taxon:** scientificNameID: urn:lsid:zoobank.org:pub:6D47380F-55E5-47CA-B692-8F6176477FB0; scientificName: *Bathynomusparacelensis*; kingdom: Animalia; phylum: Arthropoda; class: Malacostraca; order: Isopoda; family: Cirolanidae; genus: Bathynomus; **Location:** higherGeography: South China Sea; waterBody: South China Sea; islandGroup: Paracel Islands; island: Paracel Island; country: Taiwan; countryCode: Taiwan/TW; county: Taiwan; verbatimDepth: 300-550 m; minimumDepthInMeters: 300; maximumDepthInMeters: 550; verbatimCoordinates: 19.08333N, 115.25E; **Identification:** identifiedBy: Ming-Chih Huang; dateIdentified: 2024-04-28; **Geological context:** earliestEonOrLowestEonothem: Miocene; latestEonOrHighestEonothem: Miocene; **Event:** samplingProtocol: bottom trawl; year: 2023; month: 1; day: 28; verbatimEventDate: 2023-01-28; habitat: Continental slope; **Record Level:** type: PhysicalObject; modified: 2023-01-28; language: en; rightsHolder: National Museum of Marine Biology, Checheng, Taiwan; accessRights: not-for-profit use only; bibliographicCitation: *Bathynomusparacelensis* (NMMB-CD006300) for a specimen; institutionCode: National Museum of Marine Biology (NMMB); ownerInstitutionCode: NMMB; basisOfRecord: PreservedSpecimen
- **Type status:** Paratype. **Occurrence:** occurrenceRemarks: bottom trawl by the crew of Keelung-based fishing vessel Jing yang; recordedBy: Ming-Chih Huang; sex: female; lifeStage: adult; reproductiveCondition: embryos; associatedSequences: Gene Bank: PP715921, PP719187; occurrenceID: E42C574E-7C12-5F96-A847-BA305D429F0F; **Taxon:** scientificName: *Bathynomusparacelensis*; kingdom: Animalia; phylum: Arthropoda; class: Malacostraca; order: Isopoda; family: Cirolanidae; genus: Bathynomus; **Location:** waterBody: South China sea; islandGroup: Paracel Islands; island: Paracel Island; country: Taiwan; countryCode: Taiwan/TW; county: Taiwan; verbatimDepth: 300-550; minimumDepthInMeters: 300; maximumDepthInMeters: 550; verbatimCoordinates: 19.08333N, 115.25E; verbatimLatitude: 19.08333N; verbatimLongitude: 115.25E; **Identification:** identifiedBy: Ming-Chih Huang; dateIdentified: 2024-4-28; **Geological context:** earliestEraOrLowestErathem: miocene; **Event:** samplingProtocol: bottom trawl; eventDate: 2023-1-28; startDayOfYear: 2023; year: 2023; month: 1; day: 28; habitat: continental slope; **Record Level:** type: PhysicalObject, ovigerous; modified: 2023-01-28; language: en; rightsHolder: National Museum of Marine Biology, Checheng, Taiwan; accessRights: not-for-profit use only; bibliographicCitation: *Bathynomusparacelensis* (NMMB-CD006299) for a specimen; institutionCode: National Museum of Marine Biology (NMMB); basisOfRecord: PreservedSpecimen
- **Type status:** Paratype. **Occurrence:** occurrenceRemarks: bottom trawl by the crew of Keelung-based fishing vessel Jing yang; recordedBy: Ming-Chih Huang; sex: female; lifeStage: adult; preparations: whole animal; associatedSequences: Gene Bank: PP715923, PP719189; occurrenceID: 2EC78856-F8BA-5D36-89E4-0A8C300783D4; **Taxon:** scientificName: *Bathynomusparacelensis*; kingdom: Animalia; phylum: Arthropoda; class: Malacostraca; order: Isopoda; family: Cirolanidae; genus: Bathynomus; **Location:** waterBody: South China Sea; islandGroup: Paracel Islands; island: Paracel Island; countryCode: Taiwan/TW; county: Taiwan; verbatimDepth: 300-550 m; minimumDepthInMeters: 300; maximumDepthInMeters: 550; verbatimCoordinates: 19.08333 N, 115.25 E; verbatimLatitude: 19.08333 N; verbatimLongitude: 115.25 E; **Identification:** identifiedBy: Ming-Chih Huang; dateIdentified: 2024-4-28; **Geological context:** earliestEraOrLowestErathem: miocene; **Event:** eventDate: 2023-1-28; year: 2023; month: 1; day: 28; habitat: continetal slope; **Record Level:** type: PhysicalObject; modified: 2023-01-28; language: en; rightsHolder: National Museum of Marine Biology, Checheng, Taiwan; accessRights: not-for-profit use only; bibliographicCitation: *Bathynomusparacelensis* (NMMB-CD006301) for a specimen; institutionCode: National Museum of Marine Biology (NMMB); basisOfRecord: PreservedSpecimen
- **Type status:** Paratype. **Occurrence:** occurrenceRemarks: bottom trawl by the crew of Keelung-based fishing vessel Jing yang; recordedBy: Ming-Chih Huang; sex: female; lifeStage: adult; reproductiveCondition: embryos; preparations: whole animal; associatedSequences: Gene Bank: PP715924, PP719188; occurrenceID: 6776C362-B2F0-5C41-9E0F-14C6D66A1A28; **Taxon:** scientificName: *Bathynomusparacelensis*; kingdom: Animalia; phylum: Arthropoda; class: Malacostraca; order: Isopoda; family: Cirolanidae; genus: Bathynomus; **Location:** waterBody: South China Sea; islandGroup: Paracel Islands; island: Paracel Island; country: Taiwan; countryCode: Taiwan/TW; county: Taiwan; verbatimDepth: 300-550 m; minimumDepthInMeters: 300; maximumDepthInMeters: 550; verbatimLatitude: 19.08333N; verbatimLongitude: 115.25E; **Identification:** identifiedBy: Ming-Chih Huang; **Geological context:** earliestEraOrLowestErathem: miocene; **Event:** eventDate: 2023-1-28; startDayOfYear: 2023; year: 2023; month: 1; day: 28; habitat: continental slope; **Record Level:** type: PhysicalObject; modified: 2023-01-28; language: en; rightsHolder: National Museum of Marine Biology, Checheng, Taiwan; accessRights: not-for-profit use only; bibliographicCitation: *Bathynomusparacelensis* (NMMB-CD006302) for a specimen; institutionCode: National Museum of Marine Biology (NMMB); basisOfRecord: PreservedSpecimen

#### Description

**Ovum and samples** Two ovigerous individuals (NMMB-CD006299-006302). Ovum 12-15 mm in diameter (Fig. 1), slightly oval, transparent to white, without a hard shell and the soft membrane gelatinous. Some samples may be incomplete due to bottom trawl capture. The right pereonites 5-6 of NMMB-CD006299 are incomplete, the head of NMMB-CD006301 is cracked and the shapes of NMMB-CD006300 and NMMB-CD006302 are complete; see Table 1 for detailed data.

**Figure 2.**
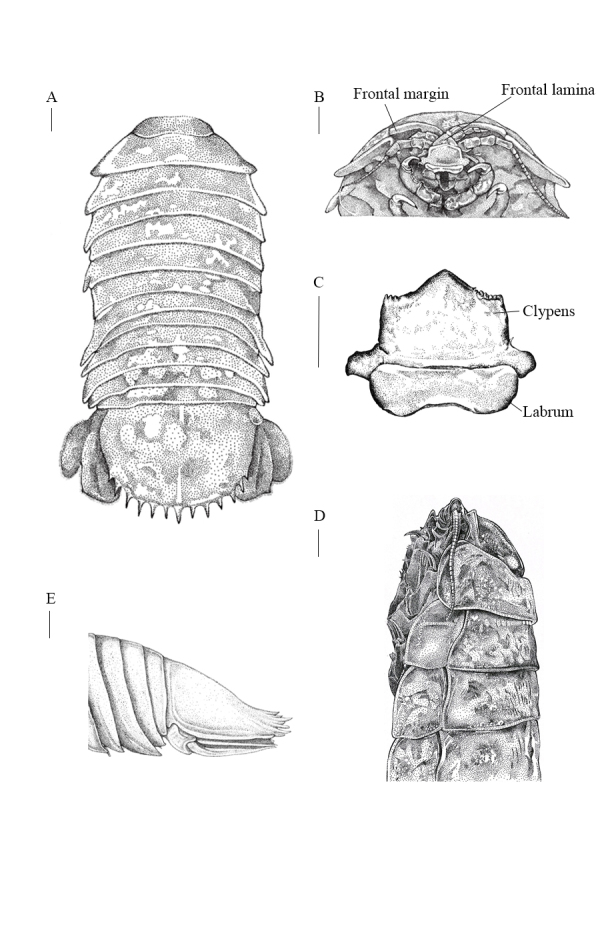
*Bathynomusparacelensis* sp. nov. holotype female (NMMB-CD006300). **A** dorsal view; **B** cephalon, anterior view; **C** clypeal region; **D** body, lateral view; **E** pleotelson, lateral view. Scale bars: 1 cm.

**Figure 3.**
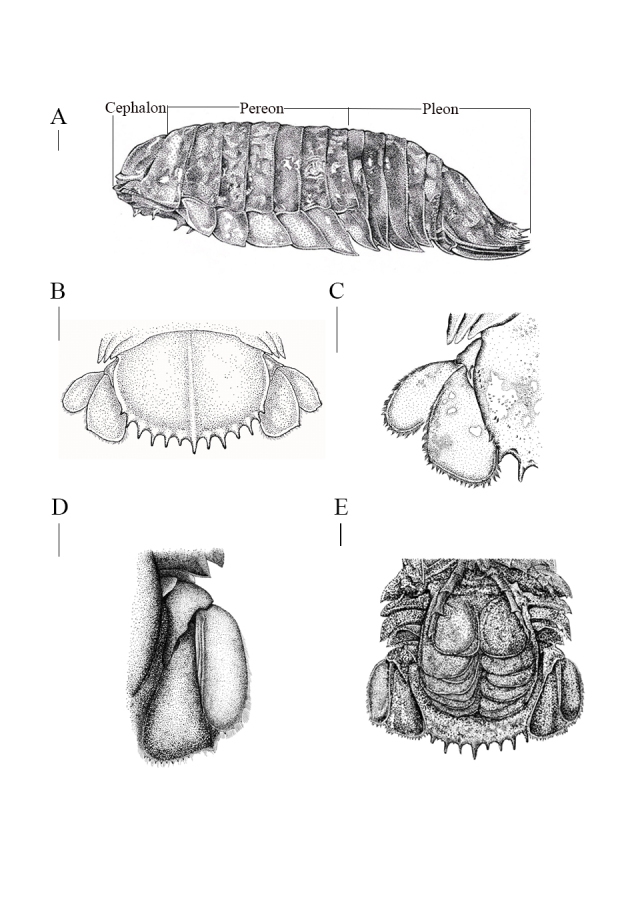
*Bathynomusparacelensis* sp. nov. holotype female (NMMB-CD006300). **A** body lateral view; **B** uropod open view; **C** uropod dorsal view; **D** uropod ventral view; **E** pleotelson ventral view. Scale bars: 1 cm.

**Female holotype** (singular numbers NMMB-CD006300, NCBI Accession No. PP715922 for *COI*, Fig. 1 and Fig. 2A). Body sub-parallel, coarsely punctate, without sculpting (Fig. 2A), total length (220 mm) approximately 2.2 times as long as maximal width (101 mm) (Fig. 1 and Fig. 2A). Cephalon (Fig. 3A) without rostral process; eyes lateral, not visible in dorsal view (Fig. 2B and E). Head with ridge above eyes discontinuous (Fig. 2B). Frontal lamina triangular, obscured in ventral view, joining cephalon, separating antennular bases; clypeus sessile, with prominent longitudinal carina, ventral margins almost straight, dorsally triangular (Fig. 2C); clypeal region distal margin slightly concave; lateral margins parallel; apex narrowly round; apex angle obtuse (about 105 degrees).

**Figure 4.**
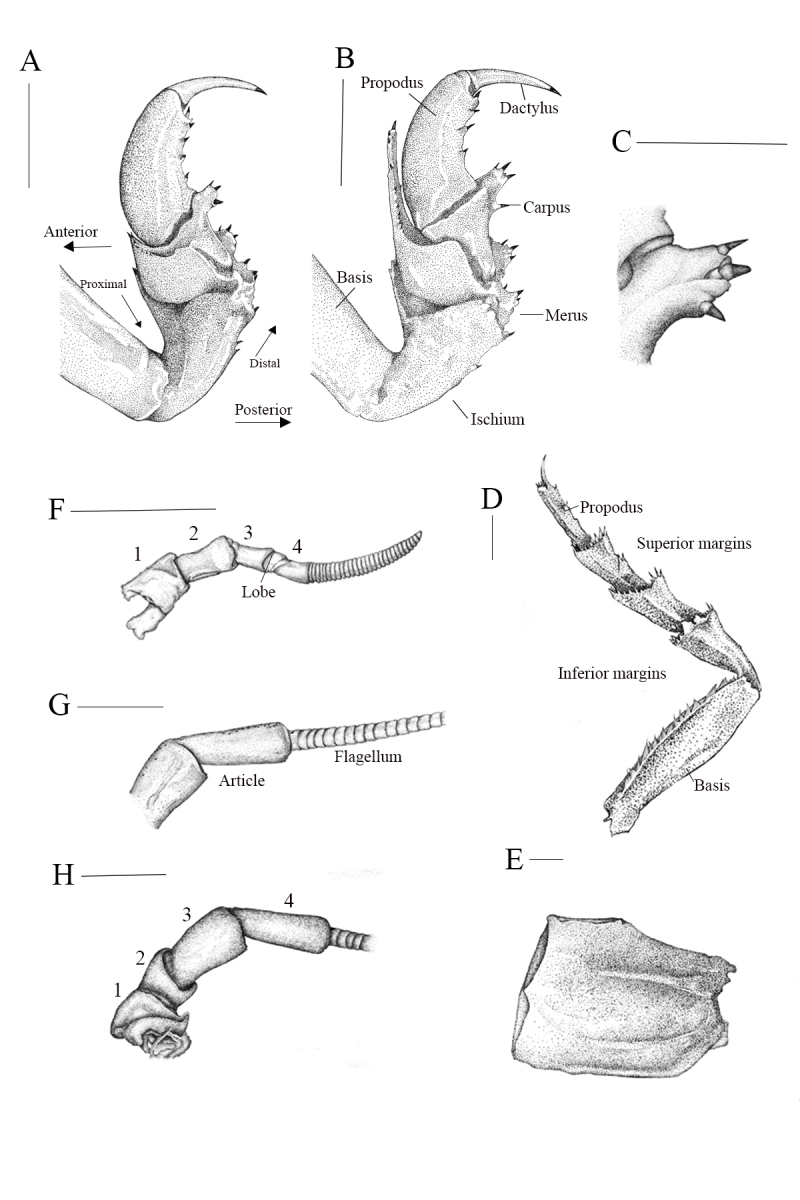
*Bathynomusparacelensis* sp. nov. (NMMB-CD006300). **A** pereopod 1, mesial view; **B** pereopod 2, mesial view; **C** pereopod 2 merus, posterolateral margin; **D** pereopod 7; **E** oostegite of pereopod 2; **F** antennula; **G**, **H** region of antennal peduncle articles. Scale bars: A, B, D-H, 1 cm, C, 0.5 cm.

Antennula peduncle 4-articulate (Fig. 4F), with a tiny lobe at the end of peduncular article 3 (Fig. 4F, lobe); articles 1 and 2 articulated; article 2 as long as article 1; articles 3 and 4 almost as long as combined lengths of articles 1 and 2; peduncle articles 1-3 almost equal in length; flagellum longer than peduncle, multi-articulate, of approximately 30 articles. Antenna peduncle 4-articulate (Fig. 4H), article 4 three times as long as wide, 1.2 times as long as article 3; article 2 the same length as article 1 (Fig. 4H); flagellum longer than peduncle and extending to the end of pleonite 2 (Fig. 2D and Fig. 3A), each section of flagellum article is longer than articles of antennula, composed of approximately 60 articles.

Mandibles (Fig. 6D), symmetrical. Incisor process of the mandible with 3 prominent black keratinised teeth on anterolateral margin (Fig. 6C); palp 3-articulate, not reaching to incisor margin (Fig. 6C). Maxilla with long setae (Fig. 6E); lateral lobe with 11 keratinised spines, 2 small individual RS, 4 RS on medial lobe (Figs. 6E and F). Maxilliped palp (Figs. 6A and B) articles broad, wider than their articulating junctions, terminal article triangular, maxilliped endite cylindrical, with rounded distal end; medial margin provided with 5 coupling hooks.

Pereonite 1 distinctly longer than other pereonites, all coxae visible in dorsal view, all with oblique carina. Coxa of pereonite 7 distally broadened and slightly curved posteriorly (Fig. 2A, Fig. 3A and E).

Pereopod 1 basis 3.2 times as long as greatest width; ischium 0.43 times as long as basis, bearing 2 posteroproximal RS and 1 RS on posterodistal margin; merus with 7 short RS on anterodistal angle, proximal row of 3 RS on posterolateral margin; carpus inferior distal margin with 3 RS; propodus approximately 2.3 times as long as maximal width, posterior margin with 5 RS (Fig. 4A), dactylus 0.52 times as long as propodus. Pereopod 2 (Fig. 4B) ischium with 2 RS each on posterior and posterodistal margins; merus with 10 short RS on anterodistal angle, posteromedial margin with 3 RS in proximal row and 3 RS in distal row: propodus approximately 2.2 times as long as maximal width, with 4 RS on posterior margin (Fig. 4B). Pereopods 3 with anterodistal margin of merus strongly produced. Pereopods 4-6 similar, becoming progressively longer towards the posterior; Pereopod 4 intermediate between Pereopod 3 and Pereopod 5. Pereopod 6 similar to pereopod 7. Pereopod 7 (Fig. 4D) basis 3.8 times as long as greatest width, superior margin convex, inferior margin with 5 palmate setae; ischium 0.58 times as long as basis, superior distal angle with 8 RS, inferior distal angle with 6 RS; merus 0.65 as long as ischium, 1.4 times as long as wide, superior distal angle with 13 RS, inferior distal angle with 8 RS; carpus 0.75 as long as ischium, 1.2 times as long as wide, inferior margin with 2 RS, superior distal angle with 10 RS, inferior distal angle with 11 RS; propodus 0.95 as long as ischium, 4.75 times as long as wide, inferior margin with 4 RS (as 1 + 2 + 1), superior distal angle with 1 RS, inferior distal angle with 6 RS; dactylus 0.42 as long as propodus.

Oostegites arising from proximal parts of pereopods 1-6 (coxae) (Fig. 4E).

Pleon (88 mm) approximately 43% of body length (220 mm) (Fig. 2A and Fig. 3A). Pleon as wide as pereon (Fig. 1 and Fig. 2A); pleonite 1 with lateral margins not produced; pleonite 2 with lateral margins produced, posterolateral angles of pleonites 3–4 reaching to almost same level posteriorly (Fig. 2A and Fig. 3A). Pleonite 3 not extending beyond pleonite 5 (Fig. 3A). Posterolateral angle of pleonites 3 and 4 extending to pleonite 5 posterior margin (Fig. 2A and Fig. 3A).

**Figure 5.**
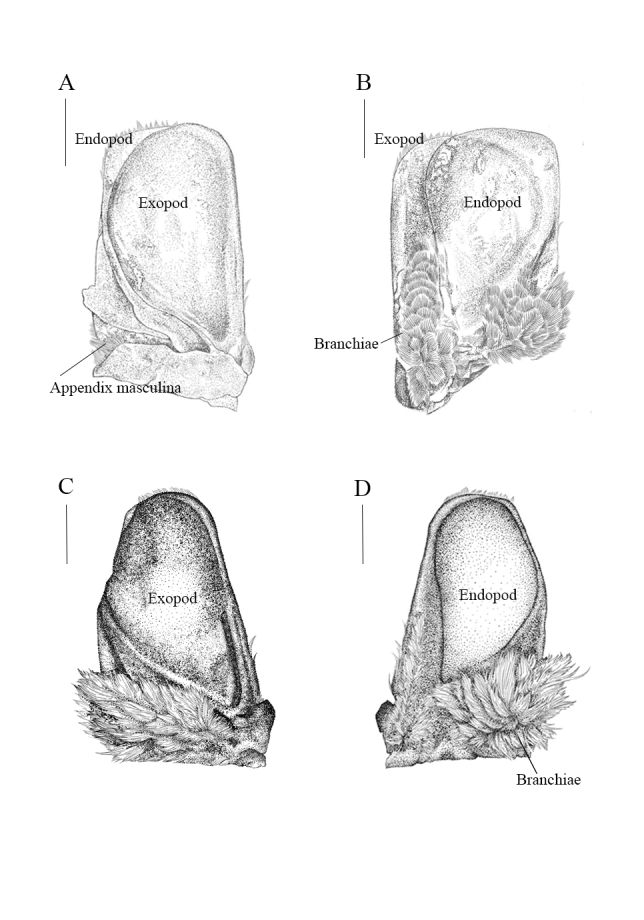
*Bathynomusparacelensis* sp. nov. (NMMB-CD006300). **A** pleopod 1, ventral view; **B** pleopod 1, dorsal view; **C** pleopod 2, ventral view, **D** pleopod 2, dorsal view. Scale bars: 1 cm.

Pleopods (Fig. 3E and Fig. 5A-D) with respiratory branchiae on dorsal and ventral surfaces of all endopods, except only ventrally on pleopod 1. Pleopods translucent, granular-surfaced film (Fig. 5). Pleopod 1 (Fig. 5A and B) exopod 1.86 times as long as wide, lateral margin straight, distally vast rounded, mesial margin strongly convex; endopod 1.52 times as long as vast, distally broadly rounded, lateral margin sinuate. Pleopod 2 (Fig. 5C and D) slightly small than pleopod 1 appearing subtriangular.

Uropodal rami not extending beyond pleotelson (Fig. 2A and Fig. 3B), peduncle with 2 ventrolateral RS: exopod and endopod with sinuate lateral and distal margins; exopod lateral margin sinuate, setal fringe of medium length (approximately 65%), with 4 RS, mesial margin straight (Fig. 3C and D), distomesial margin rounded, distal margins with 4 RS, distolateral angle slightly produced, subacute; endopod lateral margin sinuate, with 3 RS, mesial margin straight (Fig. 3C and D), distomedial angle rounded, distal margin slightly sinuate, with 9 RS, distolateral corner slightly produced, subacute (Fig. 2A, Fig. 3C and D). Pleotelson (43 mm) approximately 20% of body length, approximately 0.52 as long as wide, dorsal surface granular, with median carina; posterior margin with 11 stout, almost straight spines, plus 2 small lateral spines, others subequal, central spine not bifid; marginal setae present between spines (Fig. 2A and E).

#### Diagnosis

**Figure 6.**
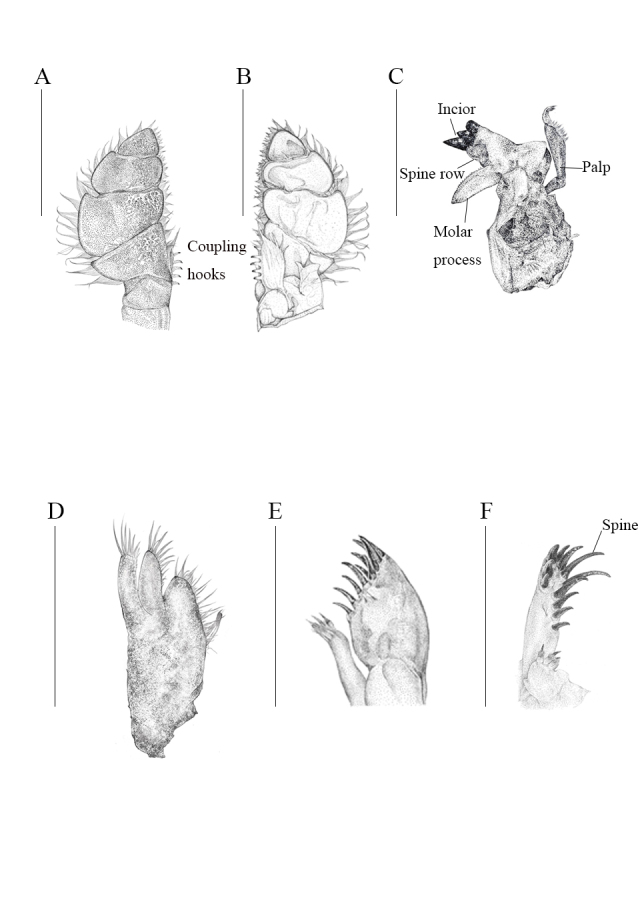
*Bathynomusparacelensis* sp. nov. (NMMB-CD006300) mouth parts. **A** right maxilliped palp (ventral); **B** right maxilliped palp (dorsal); **C** right maxilla; **D** right mandible; **E** lateral lobe of right maxilla; **F** medial view of right maxilla. Scale bars: 1 cm.

Body shape sub-parallel (Fig. 1 and Fig. 2A), body size medium, clypeus apex narrowly round, clypeus apex obtuse angle (Fig. 2C), five of maxilliped endite coupling (Fig. 6A and B), 11 of maxillula keratinised spine (Fig. 6E and F), 12 or 13 almost straight pleotelson spines (Fig. 2A, E and Fig. 3B), uropodal endopod distolateral corner slightly produced, subacute (Fig. 3B, C and D), uropodal exopod distolateral corner slightly produced, subacute (Fig. 3B, C and D), RS of uropodal endopod distributed near distomedial corner (Fig. 3B, C and D)

##### Note

The difference between *B.paracelensis* sp. nov. and *B.jamesi* includes body length (average 220 mm vs. 323 mm, Table 3), body shape (sub-parallel vs. ovate), clypeus lateral margins (parallel vs. concave), number of maxillula keratinised spine (11 vs. 9) and pleotelson spines shape (almost straight vs. upwardly curved). In *B.paracelensis* sp. nov. uropodal endopod, the anterolateral margin ends in a small tooth and the distal margin is convex. In *B.jamesi*, the anterolateral margin ends at a right angle and the distal margin is truncated.

The differences between *B.paracelensis* sp. nov. and *B.vaderi* include body length (average 220 mm vs. 279 mm), body shape (sub-parallel vs. ovate), clypeus apex shape (narrowly round vs. narrowly subacute), clypeus apex angle (obtuse angle vs. acute angle), number of maxilliped endite coupling hooks (5 vs. 4), number of maxillula keratinised spines (11 vs. 9) and pleotelson spines shape (almost straight vs. upwardly curved).

In *B.paracelensis* sp. nov. uropodal endopod, RS is mainly distributed in the distomedial margin of the endopod distal margin, with little or no RS distribution distolateral (Fig. 3C and D). This differs from *B.jamesi* or *B.vaderi*, where RS is evenly distributed in the endopod distal margin. However, this phenomenon does not exist in exopod. In addition, *B.paracelensis* sp. nov. is darker than *B.jamesi* and *B.vaderi* and closer to *B.doederleini* in body colour.

##### Variation

Specimens (NMMB-CD006299-006302): variation appears in body length/width ratio (2.16-2.26), pleotelsonic length/width ratio (0.51-0.62) and number of pleotelsonic spines (12 or 13) (Table 1). The smallest body length/width ratio is NMMB-CD006301 (2.16). On the other hand, NMMB-CD006299 with the largest (2.26). The pleotelsonic length/width ratio is also different, with NMMB-CD006299 being the largest at 0.62 and NMMB-CD006300 being the smallest at 0.51. Most of the number of pleotelsonic spines is 13, but for NMMB-CD006301, it is 12 (Table 1).

##### Molecular biology

**Figure 7.**
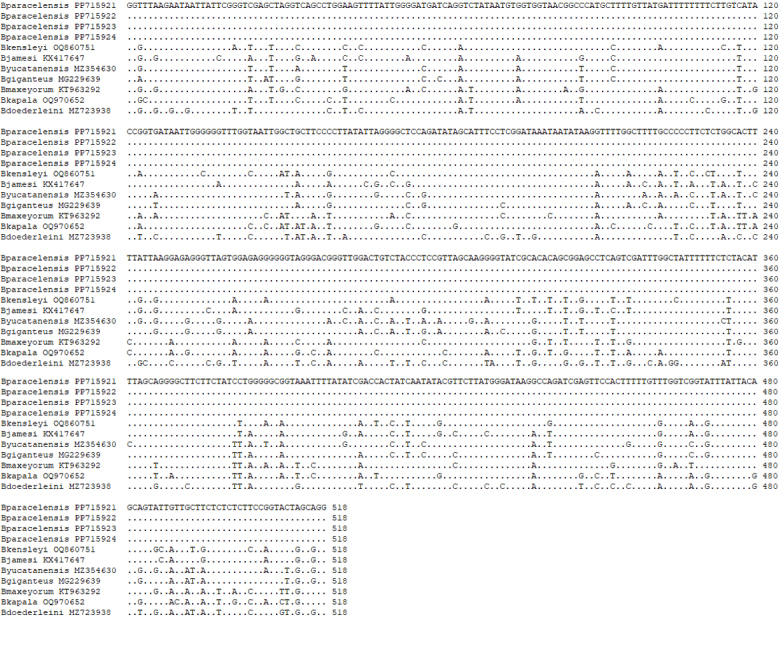
Alignment of the partial DNA sequence of the cytochrome *c* oxidase I from *Bathynomus* spp. There are four *B.paracelensis* sp. nov. (NCBI Accession Nos. PP716921, PP16922, PP16923 and PP16924), *B.kensleyi* (MZ723938), *B.jamesi* (KX417647), *B.yucatanensis* (MZ354630), *B.giganteus* (MG229639), *B.maxeyorum* (KT963292), *B.kapala* (OQ970652) and *B.doederleini* (MZ723938) .

**Figure 8.**
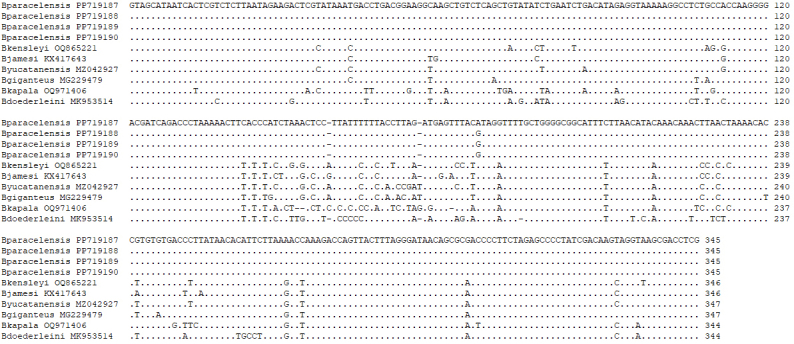
Alignment of the partial DNA sequence of the 16S rRNA from *Bathynomus* spp. There are four *B.paracelensis* sp. nov. (NCBI Accession Nos. PP719187, PP719188, PP719189 and PP719190), *B.kensleyi* (OQ865221), *B.jamesi* (KX417643), *B.yucatanensis* (MZ042927), *B.giganteus* (MG229479), *B.kapala* (OQ971406) and *B.doederleini* (MK953514).

Amplified PCR products of 518 bp from *COI* and 508 bp from 16S rRNA, respectively, were obtained for the *COI* and 16S rRNA nucleotide sequences of four specimens *B.paracelensis* sp. nov. (NMMB-CD006299-006302), respectively (Fig. 7, Fig. 8 and Table 1). The results of *COI* and 16S rRNA alignments showed that samples collected from the Paracel Islands are various from *B.jamesi* and *B.doederleini* (Fig. 7 and Fig. 8). The sequence data have been submitted to DDBJ/EMBL/GenBank on 28 April 2024 (New Paracel *Bathynomus* (NPB), Accession Nos. PP715921 (NPB1, NMMB-CD006299), PP715922 (NPB2, NMMB006300), PP715923 (NPB3, NMMB-CD006301) and PP715924 (NPB4, NMMB-CD006302) for *COI* and PP719187 (NPB1, NMMB-CD006299), PP719190 (NPB2, NMMB-CD006300), PP719189 (NPB3, NMMB-CD006301) and PP719188 (NPB4, NMMB-CD006302) for 16S rRNA. The four individuals are identical in the analysed 518-bp *COI* gene sequences (Fig. 7) and 508 bp (Fig. 8, only 345 bp are shown). The *COI* sequences of the four individuals are all the same. On the other hand, 16S rRNA sequences also show a high degree of identity, with only one base pair variation in the 508-bp 16S rRNA gene sequences. This base pair variation exists in sample NMMB-CD006299 at position ca. 270 G > A (PP719187) (Fig. 8).

The *COI*s sequence of all *Bathynomus* species currently submitted in the NCBI database is used for DNA sequence comparison. Species include *B.kensleyi* (MZ723938), *B.jamesi* (KX417647), *B.yucatanensis* (MZ354630), *B.giganteus* (MG229639), *B.maxeyorum* (KT963292), *B.kapala* (OQ970652), and *B.doederleini* (MZ723938). The comparison results show that the differences between *B.paracelensis* sp. nov. and other species are as follows: *B.kensleyi* (65 bases different, base different ratios 12.5%), *B.jamesi* (70, 13.5%), *B.yucatanensis* (74, 14.3%), *B.giganteus* (66, 12.7%), *B.maxeyorum* (75, 14.5%), *B.kapala* (89, 17.2%) and *B.doederleini* (101, 19.5%). It can be seen from the base that there are different ratios of more than 12.5%, indicating that it may not be the same species.

On the other hand, 16S rRNA sequences (345 bp) are also compared. Results with a high rate of variation can be obtained (Fig. 8). The comparison results show that the differences between *B.paracelensis* sp. nov. and other species are as follows: *B.kensleyi* (37, 10.7%), *B.jamesi* (32, 9.3%), *B.yucatanensis* (34, 9.9%), *B.giganteus* (31, 9.0%), *B.kapala* (55, 15.9%) and *B.doederleini* (55, 15.9%).

After *COI* and 16S rRNA sequencing and comparison with an NCBI databank, the results confirmed that four specimens were all new sequences and the sequences do not exist in the NCBI database.

#### Etymology

The epithet is an adjective derived from the name of the nearest island to the point of collection, the Paracel Islands. The Japanese name: Seisagusokumushi.

#### Distribution

The samples were captured in the South China Sea and the closest island is Paracel Island.

#### Taxon discussion

The clypeus of *Bathynomus* is polymorphic, including lateral margins that are parallel or concave, distal margins that are straight or concave and an apex shape and angle. *B.paracelensis* sp. nov. exhibits an obtuse angle in the apex (about 105 degrees) (Fig. 2C is very different from the acute angle in *B.vaderi* (about 70 degrees) (Ng et al. (2025), figs. 5C and 6C). In addition, there is also a difference in whether the ends of the lateral margins on both sides are sharp. It is sharp in *B.vaderi* (Ng et al. 2025), but not in *B.paracelensis* sp. nov. and *B.jamesi*. The clypeus distal margin appears serrated in *B.paracelensis* sp. nov., while *B.jamesi* appears smooth and *B.vaderi* has a few serrated margins.

Uropodal endopod differences can be found between *B.paracelensis* sp. nov. and *B.jamsei*. In *B.paracelensis* sp. nov., the anterolateral margin ends in a slight tooth and the distal margin is convex (Fig. 3B, C and D). In *B.jamesi*, the anterolateral margin ends at a right angle and the distal margin truncates (Huang et al. (2022), figs. 4d and e).

The distribution of RS in the uropodal endopod distal margin is also very special, unlike the distribution of *B.jamesi* (Huang et al. (2022), figs. 4d and e) and *B.vaderi* (Ng et al. (2025), figs. 8d and e). *B.paracelensis* sp. nov. is distributed near the distomedial corner (Fig. 3C and D).

Pleotelson spines are straight or upwardly curved. Lowry and Dempsey (2006) recorded that only *B.kensleyi* and *B.lowryi* have upward curves, but now *B.jamesi* (Huang et al. 2022) and *B.vaderi* (Ng et al. 2025) have been added. Pleotelson spines in *B.paracelensis* sp. nov. are slightly upwardly curved, but not as much as in *B.jamesi* and *B.vaderi* (Fig. 2E).

In *B.doederleini* and *B.affinis*, the number of maxilulla lateral lobe with 11 keratinised spines (Shih (1972), figs. 3 and 4). However, the maxilulla spines of *B.jamesi* are only nine (Huang et al. (2022), fig. 7e).

The number of maxilulla spines is also one of the classified items. The earliest classification of maxilulla spine was *B.decemspinosus* (Shih 1972) and its scientific name is the number of maxilulla spines. In *B.paracelensis* sp. nov., the number of maxilulla lateral lobes is with 11 keratinised spines (Fig. 6E and F), but in *B.vaderi*, there are only nine (Ng et al. 2025, fig. 7e). The number of maxilliped endite coupling hooks also differs in *Bathynomus* species, with five in *B.paracelensis* sp. nov. (Fig. 6A and B). *B.jamesi* also has five roots (Huang et al. (2022), fig. 7b), but in *B.vaderi*, there are only four (Ng et al. (2025), fig. 7b).

## Analysis

### Phylogenetic analyses

**Figure 9.**
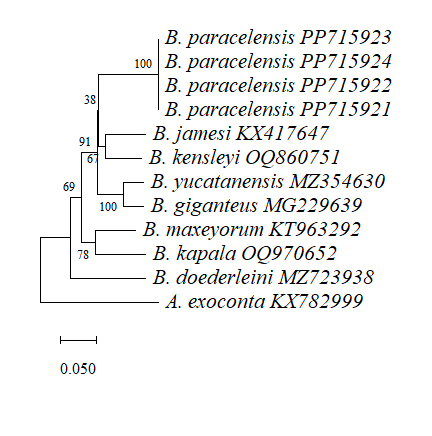
The phylogenetic tree is based on the cytochrome *c* oxidase I (*COI*) DNA sequences. The sequences were aligned using Clustal Omega and the Neighbour-joining method constructed the tree. Numbers at branches indicate bootstrap values. The sequences of Cirolanidae (*Atarbolanaexoconta*, KX782999) *COI* were used as the outgroup. Evolutionary analyses were conducted in MEGA 11.

A phylogenetic tree of *Bathynomus* was drawn using molecular evolution theory and the *COI* sequence. Fig. 9 shows the relative relationship between different species of *Bathynomus*. The closest relative of *B.paracelensis* sp. nov. in this graph is a clade comprised of *B.jamesi* and *B.kensleyi*. *B.doederleini* is more distantly related to *B.paracelensis* sp. nov.

**Figure 10.**
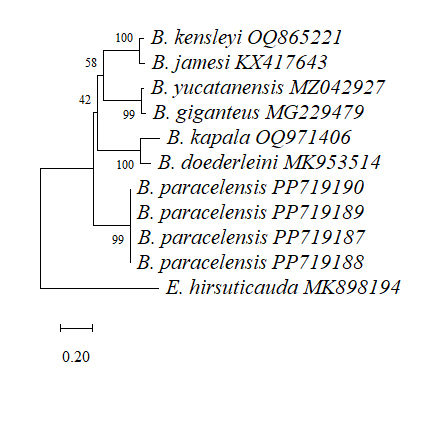
The phylogenetic tree is based on the 16S rRNA sequences. The sequences of Cirolanidae (*Excirolanahirsuticauda*, MK898194) 16S rRNA were used as the outgroup. Evolutionary analyses were conducted in MEGA 11.

Fig. 10 is a phylogenetic tree drawn, based on 16S rRNA. The nearest relative of *B.paracelensis* sp. nov. in this graph is a clade comprised of *B.doederleini* and *B.kapala*.

### Barcoding gap analysis

As shown in Table 4, the shortest pairwise distance between interspecies occurs in *B.giganteus* vs. *B.yucatanensis* (5.88%). The *B.paracelensis* sp. nov. distance to the closest related *B.kensleyi* is 14.14%, 2.4 times the shortest distance amongst known *Bathynomus* interspecies.

The average pairwise distance between *Bathynomus* interspecies calculated from Table 4 is 16.77%. The average pairwise distance of *B.paracelensis* sp. nov. for other *Bathynomus* is 17.16%, showing that the average pairwise distance of *B.paracelensis* sp. nov. is higher than the average.

Comparing *B.paracelensis* sp. nov. with other *Bathynomus*, *B.kensleyi* has the smallest pairwise distance (14.14%) and *B.doederleini* has the largest pairwise distance (23.22%), indicating that it has the closest relationship with *B.kensleyi* and the most distant relationship with *B.doederleini*. The average pairwise distance of *B.paracelensis* sp. nov. for other *Bathynomus* is 17.16%, which is greater than *B.kensleyi* (15.23%), *B.giganteus* (14.68%), *B.jamesi* (15.80%) and *B.yucatanensis* (14.70%), only smaller than *B.doederleini* (20.57%) and *B.maxeyorum* (17.42%).

*B.jamesi*, which has the closest geographical relationship, has a pairwise distance of 15.30% from *B.paracelensis* sp. nov., which is greater than *B.kensleyi* (14.14 %) and *B.giganteus* (14.31%).

## Discussion

### Morphological comparison

*B.paracelensis* sp. nov. is the smallest supergiant *Bathynomus* in the South China Sea. The body shape is similar to *B.doederleini*. *B.paracelensis* sp. nov. may be an intermediate organism between giant and supergiant *Bathynomus*.

The morphological comparison of *B.paracelensis* sp. nov. with *B.jamesi* and *B.vaderi* is shown in Table 3. Characteristic features unique to *B.paracelensis* sp. nov., such as body shape sub-parallel, clypeus apex angle, number of maxilliped endite coupling five and number of maxilliped keratinised spine number 11, were compared. The comparison shows that *B.paracelensis* sp. nov. is a newly-described species.

### Molecular diagnostics

**Alignment of DNA sequences**

The difference of DNA sequences in various *Bathynomus* is compared through DNA alignment. Fig. 7 and Fig. 8 show the comparison results of *COI* and 16S rRNA, respectively. From the DNA alignment in Fig. 7, it can be seen that the differences between *B.paracelensis* sp. nov. and other *Bathynomus* bases are: *B.jamesi* (70 bases different, base different ratios 13.5%), *B.kensleyi* (65, 12.5%), *B.yucatanensis* (74, 14.3%), *B.giganteus* (66, 12.7%), *B.maxeyorum* (75, 14.5%), *B.kapala* (89, 17.2%) and *B.doederleini* (101, 19.5%). The data show that the species with the greatest difference from *B.paracelensis* sp. nov. is *B.doederleini* (19.5%) and the least difference is *B.kensleyi* (12.5%). It is shown that, amongst the known *Bathynomus*, the closest relative to *B.paracelensis* sp. nov. is *B.kensleyi*.

**Phylogenetic tree analysis**

Phylogenetic trees, based on *COI* and 16S rRNA sequences (Figs 9, 10), showed different results. *B.paracelensis* sp. nov. was closer to supergiant *B.kensleyi and B.jamesi* in the analysis using *COI* as the marker. However, it was closer to giant *B.doederleini* and *B.kapala* in the analysis using 16S rRNA as the marker.

One of the reasons for this opposite result may be that there are fewer species and the selected DNA length is too short in the 16S rRNA sequence. Due to differences in length and location, there are limitations on the common lengths that can be compared. Compared with *COI*, 16S rRNA has fewer species (six species) and a comparable DNA length (345 bp). Another possibility is that *B.paracelensis* sp. nov. may be a *Bathynomus* species between supergiant and giant.

**Barcoding gap analysis**

Since the *COI* sequences of the four *B.paracelensis* sp. nov. are precisely the same and there is no base variation (Fig. 7), the DNA barcoding gap of intraspecies cannot be calculated, so only the pairwise distance of interspecies can be calculated.

The two data confirm that *B.paracelensis* sp. nov. and *B.jamesi* are different species. One is that the shortest pairwise distance amongst *Bathynomus* interspecies occurs between *B.giganteus* and *B.yucatanensis* (5.88%) (Table 4). The shortest pairwise distance between *B.paracelensis* sp. nov. is *B.kensleyi* (14.14%), 2.4 times the shortest distance amongst known *Bathynomus* interspecies. Another 16.77% of the data represents the average pairwise distance of *Bathynomus* interspecies. For *B.paracelensis* sp. nov., the average pairwise distance of *Bathynomus* is 17.16%, which is already higher than 16.77%, indicating that the possibility of its becoming an independent species is high. The above conclusion can confirm that *B.paracelensis* sp. nov. and *B.jamesi* are different species.

### Morphological and genetic test

*Bathynomus* from the South China Sea and the Indian Ocean have been misidentified (Huang et al. 2022, Huang and Bruce 2024). Since genetic identification had not yet been developed, Lowry and Dempsey (2006) identified *B.jamesi* and *B.kensleyi* as the same species. However, without a morphological basis, misidentifications may also occur. *Bathynomus* species native to the Indian Ocean were identified as *B.decemspinosus*, *B.doederleini* and *B.kensleyi* (Sankar et al. 2011, PrasannaKumar et al. 2020), which are misidentifications due to a lack of morphological basis. Since the appearance of *Bathynomus* is quite similar, in the future species identification of *Bathynomus*, both morphological and genetic testing results need to be completed, becoming a necessary condition for the birth of new species.

Morphology is a very complex science. It compares all body structures and is the basis of taxonomy. However, many individuals have morphological differences unrelated to the species, making identification prone to errors.

Compared with morphology, genetic classification is sharper. Usually, when confirming a new species, double confirmation through morphological and genetic systems is more reliable and genetics also requires more than two markers (such as *COI* and 16S rRNA) to be more stringent. *Bathynomus* without gene information is worthy of re-examination and review to confirm whether it is a misidentification caused by intra-specific differences or an error caused by two different *Bathynomus* species being too similar in morphology. This study used morphological and Barcoding gap analysis to compare the structure and genetics of *B.paracelensis* sp. and *B.jamesi* and confirmed that the two are different species. Since *B.vaderi* only has a morphological description (Ng et al. 2025) and no *COI* and 16S rRNA data for comparison, this article compares *B.paracelensis* sp. with *B.vaderi*, which can only be restricted to morphology.

## Supplementary Material

XML Treatment for Bathynomus paracelensis

## Figures and Tables

**Table 1.** Morphological characteristics vary, and the National Center for Biotechnology Information numbers of four *Bathynomusparacelensis* sp. nov. TL: total length, CL: cephalic length, PL: pleotelson length, PW: pleotelson width, Ant 2: antenna 2 flagellum extending to pereonite.

NMMB-CD00	TL (mm)	CL (mm)	TL/CL	PL (mm)	PW (mm)	PL/PW	Ant 2	Weight (g)	Spine Num	*COI* (NCBI)	16S rRNA
6299	226	100	2.26	52	84	0.62	P2	325	13	PP715921	PP719187
6300	224	101	2.22	42	82	0.51	P2	237	13	PP715922	PP719190
6301	225	104	2.16	45	85	0.53	P2	295	12	PP715923	PP719189
6302	205	93	2.20	44	75	0.59	P2	361	13	PP715924	PP719188

**Table 2.** List of primer pairs and PCR annealing temperatures (*T*m) used to amplify *COI* and 16s rRNA genes.

Primers		Sequence 5'-3'	*T*m (ﾟC)
*COI* primers (Folmer et al. 1994)			
LCO-1490 (F)		GGT CAA CAA ATC ATA AAG ATA TTG G	48
HCO-2198 (R)		TAA ACT TCA GGG TGA CCA AAA AAT CA	48
*COI* primers (our design)			
Kmae (F)		GTT GGA ACA GGG TTA AGA AT	48
Kushi (R)		AGT ATT AAG GTT GCG ATC TG	48
16S primers (Palumbi et al. 1991):			
16Sar (F)		CGC CTG TTT ATC AAA AAC AT	56
16Sbr (R)		CCG GTC TGA ACT CAG ATC ACG T	56

**Table 3.** Comparison of morphological and ecological characters amongst *Bathynomus* in the North East Pacific.

Species	B.paracelensis sp. nov.	B.jamesi*	B.vaderi**	B.doederleini***
Giant or Supergiant	Supergiant	Supergiant	Supergiant	Gaint
Body shape	Sub-parallel	Ovate	Ovate	No recorded
Body average length (sample size)	220 mm (n=4)	323 mm (n=10)	279 mm (n=6)	133 mm (n=1)
Body length/ width ratio	2.16-2.26	2.2-2.4	1.9	3
Body color	Brown pattern	Dark-yellowish-grey	Light purple (from photo)	Blood red
Clypeus: lateral margins	Parallel	Concave	Parallel	Concave
Clypeus: distal margin	Slightly concave	Slightly concave	Slightly concave	Straight
Clypeus: apex shape	Narrowly round	Narrowly round	Narrowly subacute	Wide round
Clypeus: apex angle (degrees)	105	95	70	65
Head ridge above eyes	Discontinuous	Discontinuous	Discontinuous	Discontinuous
Maxilliped endite coupling hook number	5	5	4	5
Maxillula keratinised spine number	11	9	9	11
Antennal flagellum reach	Pereonite 2	Pereonite 2	Pereonite 2	Pereonite 3
Lateral margin of pereonite: color	White	Yellow or Beige	Cream yellow	No recorded
Pereopod 7: shape of distal part of coxa	Distally narrowed	Distally broadened	Distally narrowed	No recorded
Pleotelson length/width ratio	0.51-0.62	0.6	0.6	0.9
Pleotelson spines: number	12 or 13	11 or 13	11+2	5 (occasionally 7)
Pleotelson spines: shape	Almost Straight	Upwardly curved	Upwardly curved	Straight
Pleotelson central spines: shape	Simple	Simple	Simple	Simple
Uropodal endopod: RS in distal margin	Near distomedial corner	Average	Average	Average
Uropodal endopod: distolateral corner	Slightly produced	Slightly produced	Produced	Produced
Uropodal endopod: distolateral corner	Subacute	Subacute	Acute	Subacute
Uropodal exopod: distolateral corner	Slightly produced	Produced	Produced	Slightly produced
Uropodal exopod: distolateral corner	Subacute	Subacute	Acute	Acute
Habitat depth	300-550m	420–550 m	depth not known	100-600m
Distribution	South China Sea, Paracel	South China Sea, Pratas	Offshore of Quy Nhơn City	All western Pacific Ocean
*Huang et al., 2022, **Ng et al., 2025, ***Lowry and Dempsey 2006; Shih 1972.				

**Table 4.** The pairwise distance of *COI* gene segment (596 bp) amongst studied species of *Bathynomus*. Numbers in parentheses indicate the number of individuals.

	1	2	3	4	5	6	7	8
1. *B.paracelensis* sp. nov. (4)								
2. *B.jamesi*_KX417647 (1)	0.1530							
3. *B.doederleini*_MZ723938 (1)	0.2322	0.2205						
4. *B.kensleyi*_OQ860751 (1)	0.1414	0.1076	0.2099					
5. *B.yucatanensis*_MZ354630 (1)	0.1627	0.1288	0.2030	0.1337				
6. *B.giganteus*_MG229639 (1)	0.1431	0.1270	0.2097	0.1319	0.0588			
7. *B.maxeyorum*_KT963292 (1)	0.1656	0.1706	0.2047	0.1627	0.1721	0.1677		
8. *B.kapala*_OQ970652 (1)	0.2031	0.1938	0.1866	0.1682	0.1824	0.1859	0.1677	
